# Comparative Metabolomic Profiling of the Metabolic Differences of Δ9-Tetrahydrocannabinol and Cannabidiol

**DOI:** 10.3390/molecules27217573

**Published:** 2022-11-04

**Authors:** Qianru Rao, Ting Zhang, Manyun Dai, Bin Li, Qianlun Pu, Min Zhao, Yan Cheng, Dongmei Yan, Qi Zhao, Zhanxuan E. Wu, Fei Li

**Affiliations:** 1 Laboratory of Metabolomics and Drug-Induced Liver Injury, Frontiers Science Center for Disease-Related Molecular Network, West China Hospital, Sichuan University, Chengdu 610041, China; 2 Academician Workstation, Jiangxi University of Chinese Medicine, Nanchang 330004, China; 3 States Key Laboratory of Phytochemistry and Plant Resources in West China, Kunming Institute of Botany, Chinese Academy of Sciences, Kunming 650201, China; 4 Advanced Mass Spectrometry Center, Research Core Facility, Frontiers Science Center for Disease-Related Molecular Network, West China Hospital, Sichuan University, Chengdu 610041, China

**Keywords:** cannabis, Δ9-tetrahydrocannabinol, cannabidiol, metabolomics

## Abstract

More than one hundred cannabinoids have been found in cannabis. Δ9-Tetrahydrocannabinol (THC) is the recognized addictive constituent in cannabis; however, the mechanisms underlying THC-induced toxicity remain elusive. To better understand cannabis-induced toxicity, the present study compared the metabolic pathways of THC and its isomer cannabidiol (CBD) in human and mouse liver microsomes using the metabolomic approach. Thirty-two metabolites of THC were identified, including nine undescribed metabolites. Of note, two glutathione (GSH) and two cysteine (Cys) adducts were found in THC’s metabolism. Molecular docking revealed that THC conjugates have a higher affinity with GSH and Cys than with the parent compound, THC. Human recombinant cytochrome P450 enzymes, and their corresponding chemical inhibitors, demonstrated that CYP3A4 and CYP1B1 were the primary enzymes responsible for the formation of THC-GSH and THC-Cys, thus enabling conjugation to occur. Collectively, this study systematically compared the metabolism of THC with the metabolism of CBD using the metabolomic approach, which thus highlights the critical role of metabolomics in identifying novel drug metabolites. Moreover, this study also facilitates mechanistic speculation in order to expand the knowledge of drug metabolism and safety.

## 1. Introduction

**Figure 1:**
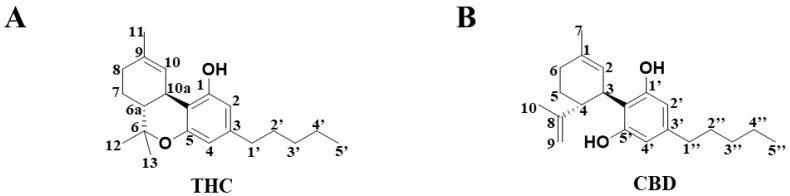
The chemical structure of compounds in (**A**) THC and (**B**) CBD.

Cannabis is an excellent natural source of fiber, and it is known to produce both a recreational effect and provide medicinal value. Δ^9^-Tetrahydrocannabinol (THC) (Figure 1A) and cannabidiol (CBD) (Figure 1B) are the two major bioactive C21 terpenophenolic compounds that are isolated from the herbal medicine, *Cannabis sativa* [1]. Although THC and CBD display similar chemical structures, their biological properties are distinct. Whereas the addictive property of marijuana is mainly attributed to THC, the beneficial pharmacological effect of marijuana is likely to be mediated by CBD, an isomer of THC. On the other hand, CBD has been shown to produce antiemetic, anti-inflammatory and antipsychotic effects [2]. Nonetheless, there have been few comparative in vitro metabolomic studies [3] that investigate the differences between the metabolism of THC and CBD to date, and metabolic characteristics that are unique to THC are yet to be determined.

A metabolomics-based study is a powerful tool with which to examine drug metabolism and toxicity [4,5,6]. In this study, we employed LC–MS-based metabolomics to gain insight into the relationship between the structure of a range of THC metabolites and the combined capacities of CB1 receptors. A total of 59 drug metabolites were identified using metabolomics-based methods in this study, including nine new unreported THC metabolites and ten new unreported CBD metabolites. The formation of glutathione (GSH) (T29, T30) and cysteine (Cys) (T31, T32), and of THC conjugates, was observed, and these conjugated molecules were shown to have the potential to bind with the CB1 receptor in this study, thus providing new insights into the regular use of cannabis (THC/CBD).

## 2. Results

### 2.1. Metabolomic Profiling of HLM and MLM Treated with THC or CBD

**Figure 2:**
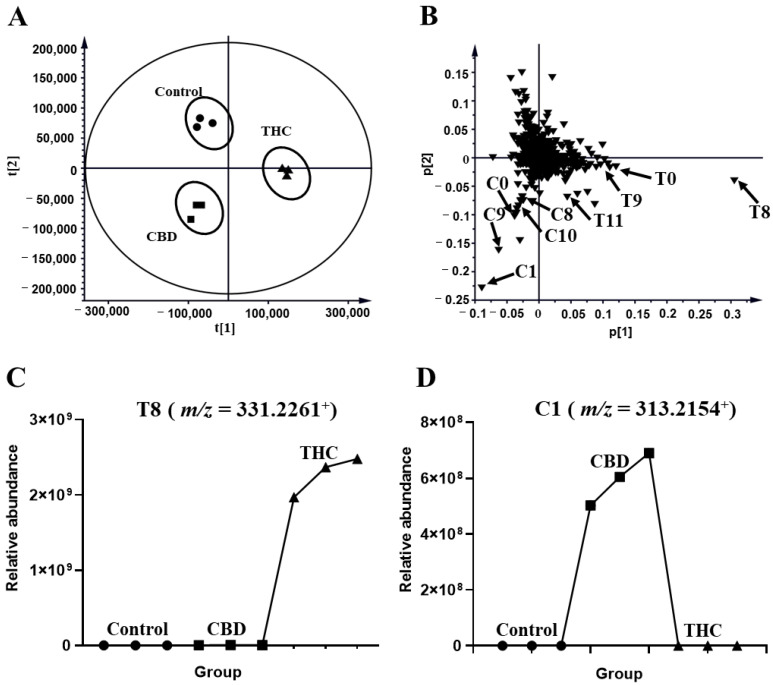
Metabolomic analysis of THC and CBD in the HLM incubation system. (**A**) Scores plot of unsupervised PCA showing the control (●), CBD (■), and THC (▲) treated HLM profiles (*n* = 3). (**B**) Loading scatter plot for the control, THC, and CBD treated HLM profiles (*n* = 3). Partial THC (T0, T4, T8–T9, T11, T17) and CBD (C0, C1, C8, C9, C10) metabolites are labeled in the Loading Scatter plot. The trend plot of **T8** (**C**) and **C10** (**D**) was showed in the control, CBD, and THC groups.

The profiles of HLM that were treated with THC and CBD, and the control group, were compared in Figure 1. The unsupervised principal analysis (PCA) model was used for the preliminary screening of metabolites that were excreted in the human liver microsome of THC and CBD metabolites (R^2^X = 0.941, Q^2^ = 0.878). Three clusters corresponded with the control, and the THC and CBD groups, thus indicating that THC and CBD could be transformed into different metabolites (Figure 2A). After certain features were selected and annotated, the distribution of the THC and CBD metabolites were validated in the loading plot. It was shown that THC and CBD metabolites were clustered in the fourth quadrant and third quadrant, respectively, thus confirming differences between the metabolism of THC and CBD (Figure 2B). The relative abundance of discriminatory metabolites was visualized with tread plots, as exemplified by T8 (*m/z* 331.2261^+^) and C10 (*m/z* 331.2264^+^), which were unique to THC-treated group and CBD-treated group, respectively (Figure 2C,D). Using this approach, metabolites of CBD and THC were also identified in the MLM profile.

### 2.2. THC and CBD Metabolites in HLM and MLM Profiles

A total of 52 phase I THC and CBD metabolites were identified. Twenty-eight metabolites in phase I were derived from THC, including 21 in HLM, and 24 in MLM (Table 1). Between the HLM and MLM profiles, in terms of THC metabolites, 17 metabolites (T1 to T12, T22 and T23, and T25 to T27) were found in two different species. The rest were found to be unique to three metabolites (T13, T14, and T17) in the HLM profile, and seven metabolites (T15, 16, T18 to T21, and T24) in the MLM profile. 

Twenty-four CBD metabolites were identified in the HLM and MLM profiles, including 14 metabolites in the HLM profile and 21 in the MLM profile (Table S1, Supplementary Materials). C1 and C2, C8–C10, C12–C15, C18, C20, and C21 were consistently found between the two systems, whereas C3, C7, and C23 were only observed in the HLM system. 

The details of the MS/MS of THC and CBD are shown in Figure S1. The formation of desaturated THC and CBD metabolites has not been described in previous studies. THC and CBD were desaturated and di-desaturated, which generated T1 and T2, and T3 to T7 metabolites, and C1 to C3, and C4 to C7 metabolites, respectively. The chemical formula of metabolites T1 and T2, C1 to C3 was C_21_H_28_O_2_, in accordance with the observed *m/z* [M + H]^+^ in 313.2129–313.2174; these values which were 2 Da (2H) lower than that of the THC and CBD parent compounds, thus indicating that these metabolites were desaturation products. The retention time of C7 was 7.85 min, thus suggesting that it was derived from the cyclization of the methyl group (C10) and the nearby hydroxyl group at C5. Other details concerning the identification and structural elucidation of these THC and CBD metabolites are provided in Figures S2 and S3 (Supplementary Materials).

### 2.3. Identification of GSH and Cys Conjugates of THC and CBD

**Figure 3:**
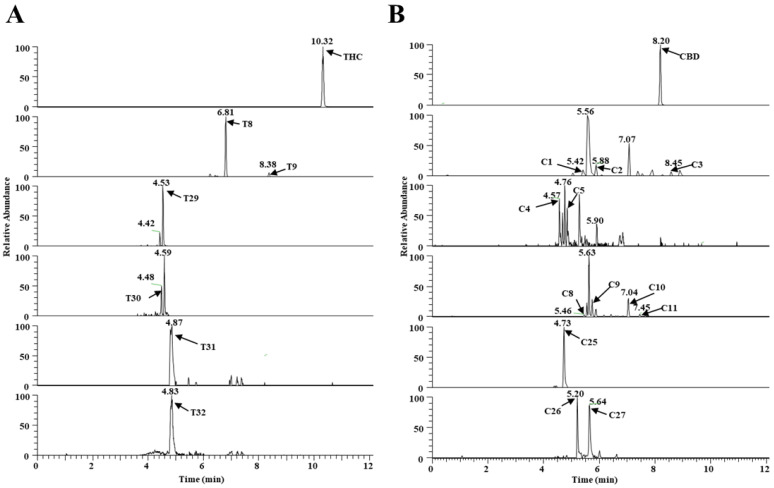
Metabolism of THC and CBD shown in a typical chromatogram of EIC, using UHPLC Q-Exactive MS in vitro. (**A**) THC metabolites (T0, T8, T9, and T29–T32) and (**B**) CBD metabolites (C1–C5, C8–C11, and C25–C27).

**Figure 4:**
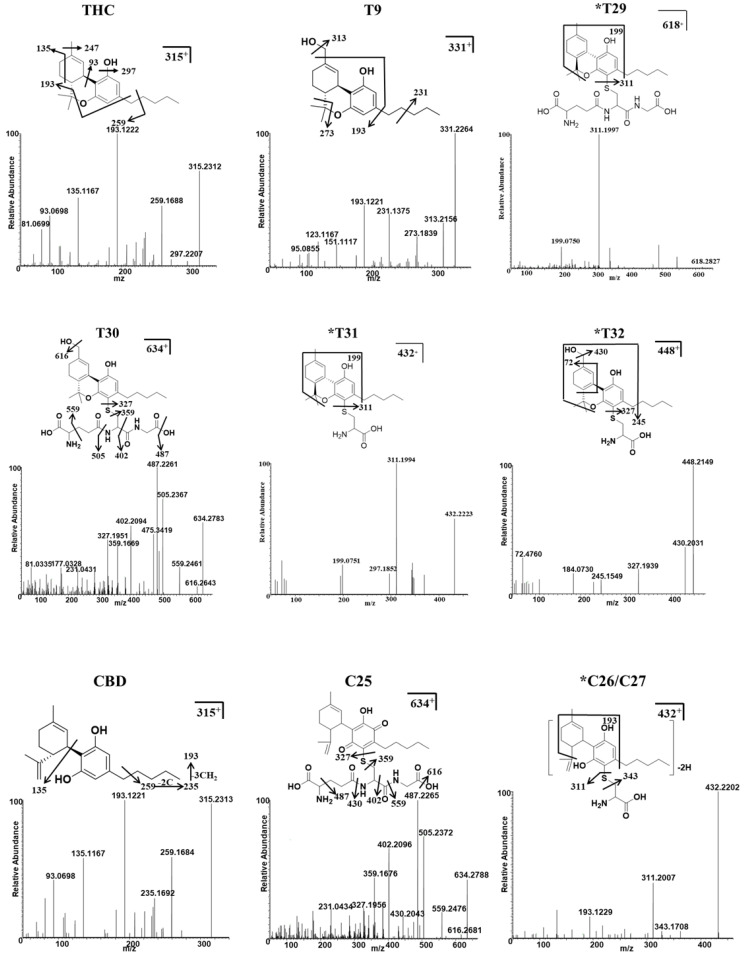
Identification of THC, CBD, and their major conjugates with GSH/Cys metabolites using UHPLC Q-Exactive MS. The MS/MS spectrum and fragmentation patterns of THC, 11-OH-THC (T9), two THC–GSH conjugates (T29–T30), two THC–Cys adducts (T31 and T32), CBD, one CBD–GSH adduct (C25), and two CBD–Cys conjugates (C26–C27). * Represents the novel metabolites in this study.

Four additional metabolites (T29–T32) that metabolize THC, and three additional metabolites (C25–C27) that metabolize CBD, were identified in the MLM and HLM profiles, and they were treated with GSH or Cys. GSH and Cys, conjugated with THC, were not dependent on NAPDH (Table 1), whereas Cys conjugated with CBD was provided by NAPDH (Table S1, Supplementary Materials). Five of these (T29, T31, and T32, and C26 and C27) were novel metabolites that have not been previously reported. The chromatogram, MS/MS spectrum, and fragmentation pathways of the representative THC and CBD metabolites are presented in Figure 3 and Figure 4.

The THC metabolites, T29 and T31, were assigned the molecular formulas C_31_H_43_N_3_O_8_S and C_31_H_43_N_3_O_9_S, based on the *m/z* ratio of the [M + H]^+^ ion at 618.2827^+^ and 634.2780^+^, respectively. The elution times of T29 and T30 were 4.53 and 4.48 min, respectively. T29 was identified as a novel GSH-conjugated metabolite, as confirmed by the presence of the MS/MS fragment and the [M + H]^+^ ion at 311.1997^+^; this was equivalent to the elimination of the GSH adduct from the parent ion. Metabolites of T31 and T32 were assigned the molecular formulas C_24_H_33_NO_4_S and C_24_H_33_NO_5_S, and they were eluted at 4.87 and 4.83 min, based on the *m/z* ratio of the protonated molecule at 432.2223^+^ and 448.2141^+^, respectively. The MS/MS spectrum of T31 also produced a major fragment with a *m/z* = 311.1994^+^, which corresponded with the loss of Cys adduct from the parent ion. The GSH/Cys adduct can be bound to the C2 or C4 position of the benzene ring of the THC metabolites. The chemical formula of the metabolite C25 was C_31_H_43_N_3_O_9_S, according to the [M + H]^+^ at *m/z* 634.2788^+^; this value was 319 Da (GSH+O-H_2_) higher than that of the CBD parent compound, thus indicating that it was a GSH-conjugated CBD metabolite in a HLM/MLM incubation system. C26 and C27 were assigned the formula C_24_H_33_NO_4_S, and they produced a major fragment with a *m/z* 311.2007^+^, which is representative of the loss of the Cys adduct from the parent ion. The positions of Cys were unable to be determined.

### 2.4. Molecular Docking Prediction of THC and CBD Metabolites

A software of Discovery Studio 2020 docking simulation was utilized to determine whether THC metabolites could activate a CB1 receptor. This tool allowed the tension between the binding energy and the active site to be resolved. The score of the -CDOCKER energy, -CDOCKER INTERACTION energy, and binding energy were used to display the molecular docking appearance. A higher -CDOCKER INTERACTION energy score indicated better binding. A negative binding energy indicated a more stable binding. When the -CDOCKER energy score was negative, it indicated a steric hindrance between receptor and ligand. THC (T0) and CBD (C0) generated a -CDOCKER INTERACTION energy score of 47.84 and 45.16, respectively (Table S5). The -CDOCKER energy was also found to be different between T0 (8.27) and C0 (−1.98). Twenty-three of the THC metabolites (T2, T7–21, and T23–32) had a higher affinity for the CB1 receptor than the THC parent compound, as shown the higher -CDOCKER INTERACTION energy score. These THC metabolites also displayed positive values for energy scores, thus suggesting that they were capable of docking into the CB1 binding pocket with minimal steric hindrance. Moreover, C2, C8, C15, C19, C23, and C24 had a higher -CDOCKER INTERACTION energy score than THC, but their CDOCKER energy scores were negative or close to zero, except for C23, thus suggesting the poor binding of these CBD metabolites due to steric hindrance.

Out of all the THC metabolites, T29–T32 were shown to be the top four metabolites with the highest -CDOCKER INTERACTION energy score (T29 = 81.27, T30 = 69.35, T31 = 55.09, and T32 = 57.20), and thus they represent the best binding modes that anchored at the active site and interacted with the key residues. On the other hand, the binding energy of T29 (THC+GSH-H_2_) was −130.96, for T30 (THC+GSH+O-H_2_) it was −17.62, for T31 (THC+Cys-H_2_) it was −47.54, and for T32 (THC+Cys+O-H_2_) it was −74.59, thus suggesting that T29 and T32 could be well docked into the binding pocket of CB1.

**Figure 5:**
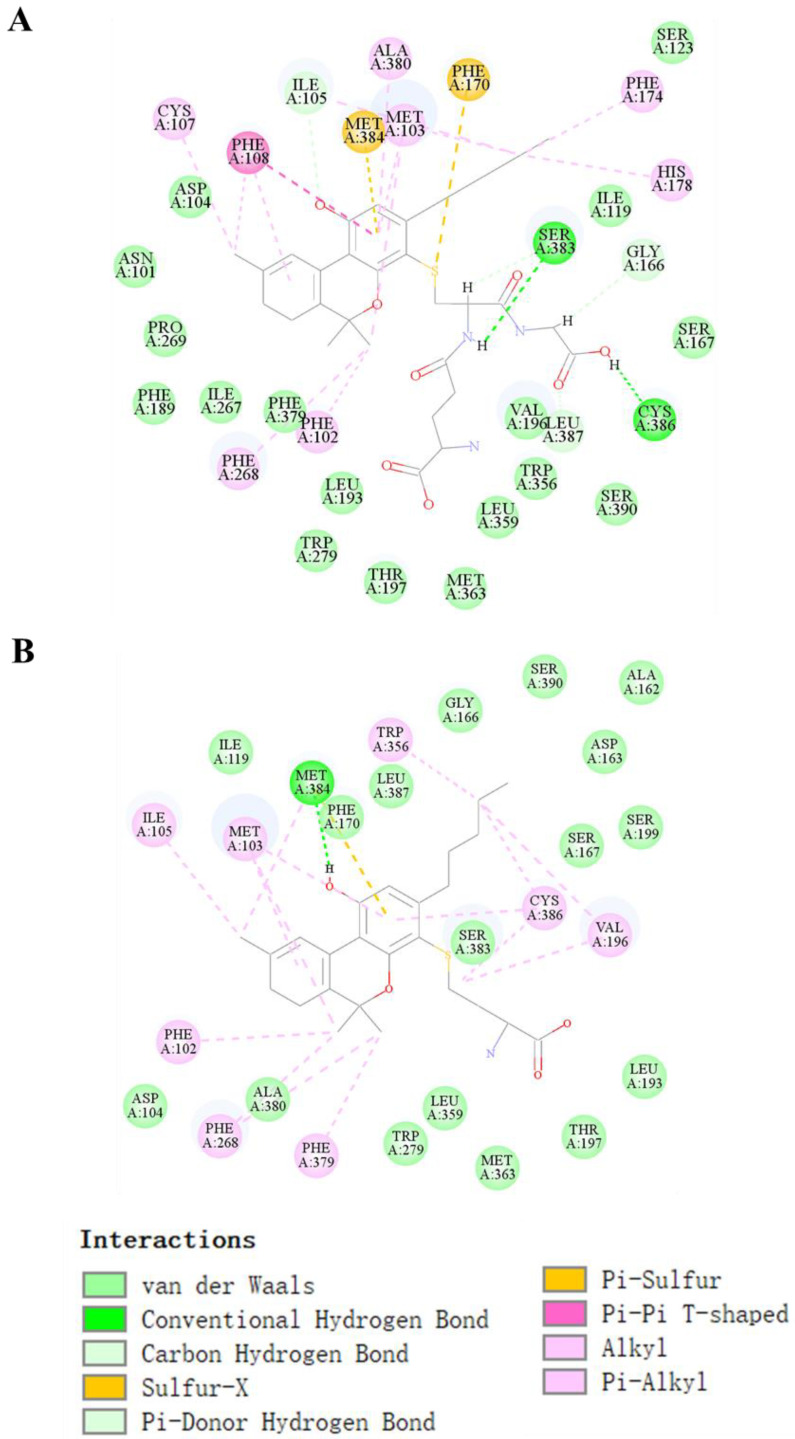
Molecular docking of THC conjugated with GSH (T29) and Cys (T31). The lowest energy docked pose and 2D interaction map in T29 (**A**) and T30; (**B**) binding site of the CB1 receptor. Active site residues involved in the interaction are represented in blue. The different colors represent the conjugated bond interactions between the target and ligand molecules.

The binding pocket of CB1 attracts THC-bound amino acid residues comprising Ala380, Ile-105, Ser-383, Met-103, Cys-386, Trp-356, Val-196, Phe-268, and Phe-108, as shown in Figure S4A (Supplementary Materials). This binding pocket also interacted with CBD through the formation of one hydrogen bond with Ser-383, six *π*-alkyl bonds with Cys-386, Leu-387, Phe-108, Pro-269, Ala-380, and Ile-105, and one *π–π* bond with Phe-268, as shown in Figure S4B. As the novel THC metabolite T29 (GSH-conjugated metabolite) had the highest affinity with the receptor, and was most stably bound to the receptor compared with the other two novel metabolites, T30–T31 (Cys-conjugated metabolites which had a similar affinity for CB1), we examined the ligand–receptor interaction more closely using T29 and T31. THC conjugated with GSH (T29) interacted with the binding pocket through the formation of two hydrogen bonds with Ser-383 and Cys-386, one *π–π* bond with Phe-108, two π-sulfur bonds with Met-384 and Phe-170, and seven π-Alkyl bonds with Cys-107, Phe-268, Phe-102, Ala-380, Met-103, Phe-174, and His-178 (Figure 5A). Such interactions facilitated the proper positioning of the compound in the active site. Many residues also participated in the molecular docking of T31 (Figure 5B), including Met-384 (H-bond), Ile-105, Met-103, Phe-102, Phe-268, Phe-379, Trp-356, Cys-386, and Val-196 (π-alkyl bond).

### 2.5. CYPs Involved in the Formation of THC and CBD Metabolites

The role of different CYPs in the formation of THC and CBD metabolites are summarized in Table 2 and Table S2. CYP3A4 is involved in the phase I metabolism of THC and CBD, and it accounts for the formation of 25 THC metabolites and 16 CBD metabolites. Among the CYPs involved in the formation of THC metabolites, CYP2C9, CYP2C19, and CYP3A4 were found to be responsible for the formation of hydroxylated metabolites. CYP2C19 and CYP3A5 were found to be the major enzymes that are required for the formation of carboxylic acid. Among the CYPs involved in the metabolism of CBD, CYP2C19, CYP2C8, and CYP2D6 were found to catalyze the formation of the hydroxylated metabolites of CBD. CYP2C19 and CYP2D6 also catalyzed the di-hydroxylation reaction of CBD.

**Figure 6:**
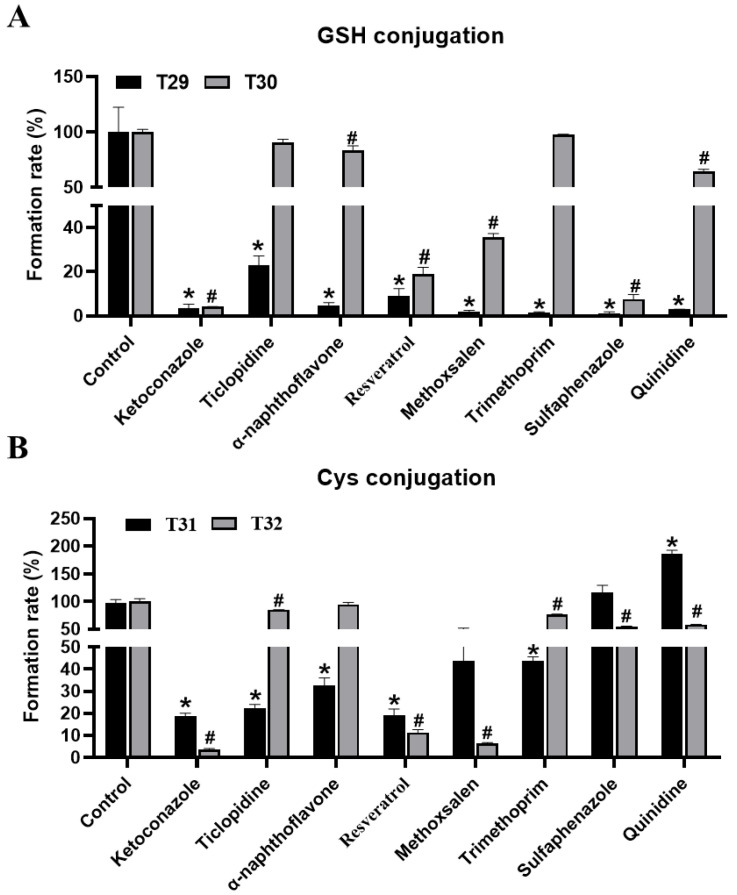
Inhibitory effects of CYP inhibitors on the formation of T29–T32 in MLM incubation systems. (**A**) Inhibitory effects of CYP inhibitors on the formation of T29 and T30 metabolites in MLM incubation systems. (**B**) Inhibitory effects of CYP inhibitors on the formation of T31 and T32 metabolites in MLM incubations systems. The relative level of abundance of each metabolite during the incubation in the MLM system without inhibitors was 100%. All the data are expressed as a mean ± SD (*n* = 3). Two group comparisons between the positive control (incubated with the parent compound without a CYP inhibitor) and each group of samples with the co-incubation of the CYP inhibitor and the parent compound was conducted using a Student’s independent t-test, followed by multiple testing corrections. * *p* < 0.05 represents the control (T29, T31) vs. other groups; # *p* < 0.05 represents the control (T30, T32) vs. other groups.

Through inhibitor assays of MLM, co-treated with THC and CYP450s inhibitors, key enzymes that are involved in the conjugation of GSH and Cys with THC were also identified. The formation of T29–T32 was suppressed to 97%, 96%, 82%, and 96% by ketoconazole at 100 μM (Figure 6A,B). Similarly, the formation of T29–T32 was suppressed up to 91%, 81%, 81% and 89% by resveratrol at 10 μM, accordingly. These results collectively suggested the pivotal role of CYP3A4, and CYP1B1 (but to a lesser extent), in the formation of the conjugated products of THC metabolites. The contribution of other CYP450s, regarding GSH-conjugated and Cys-conjugated metabolites, might be substrate- and pathway-dependent. For example, ticlopidine, α-naphthoflavone, and trimethoprim treatments led to a concordant reduction in the formation of T29 and T31, but not T30 or T32. Likewise, methoxsalen, sulfaphenazole, and quinidine significantly suppressed the formation of both GSH-conjugated products (T29–T30), but the effect was much more profound in the formation of T29 than T30. These observations suggest that the desaturated THC intermediates were the preferred substrates over the hydroxylated intermediates when processing conjugated products downstream using CYP450s. In addition, sulfaphenazole and quinidine were seen to suppress the formation of GSH-, but not Cys-conjugated products of THC, thus suggesting that the CYP450s targeted by these inhibitors might be specific for the formation of GSH-conjugated products. Taken together, CYP3A4 was the key enzyme, of primary human CYPs, that was involved in the formation of THC metabolites, and it enabled Cys and GSH to conjugate. Other CYP450s, which were involved in the adducted reaction of THC, were likely to be substrate- and pathway-dependent.

## 3. Discussion

LC-MS-based metabolomic methods have been successfully applied in order to screen for active metabolites and to find biomarkers of diseases [7]. Given the increasing prevalence of cannabis use, or the use of single components of THC over the last decade, many countries, such as Canada in 2018, have legalized non-medicinal cannabis use for adults. Moreover, several states in the United States have expanded the legalization of medicinal and recreational cannabis use in recent years [8]; however, the metabolic rate of THC, as well as the manner in which THC metabolites are linked to the psychoactive and cytotoxicity of THC, is yet to be determined.

**Figure 7:**
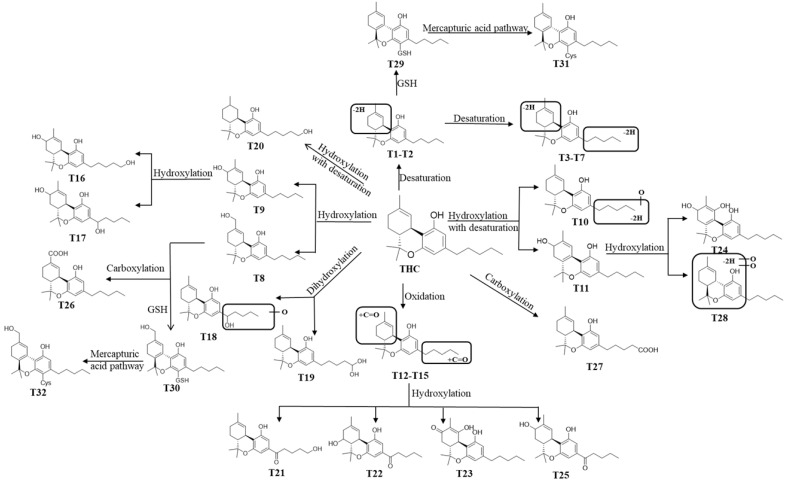
Proposed metabolic pathways of THC.

In this study, we employed a comparative pharmaco-metabolomics approach in order to profile the metabolism of THC and CBD. Numerous metabolites (including those derived from THC and CBD) were identified using LC/HRMS in cannabis extracts [9]; however, the metabolic profiles of CBD and THC are not clearly defined. Based on the comprehensive profile of THC-related metabolites, the map of the metabolic pathways of THC (Figure 7) and CBD (Figure S6) was constructed. THC was hydroxylated, desaturated, and hydroxylated with desaturation and carboxylation in order to form phase I metabolites (T1–T28), and it was reacted with GSH to form GSH/Cys conjugated metabolites (T29–T32) via mercapturic acid pathways. In accordance with previous findings which found that non-psychotropic CBD could not be transformed into psychotropic THC under non-acidic conditions [10], the conversion of CBD to THC or THC-related metabolites was not observed in any of the incubation systems tested in the present study. This implies that the C8 position of the double bond structure of CBD is stable, and forming a ring with the adjacent hydroxyl group is difficult using our systems. Through the comparison of the metabolic profiles of the metabolism of THC and CBD in vitro, we found that the metabolite rate of THC was higher than CBD in both the HLM and MLM systems. Resulting from the differences in spatial conformation of THC and CBD, THC presents a planar structure whereas CBD has a slightly angular structure. Such differences may explain the altered metabolic rate between these two compounds. Among the 21 phase I metabolites of THC that were found in HLM, 24 THC metabolites were found in MLM. Moreover, the relative abundance of downstream metabolites that were derived from THC was considered to be greater than that of CBD after a set amount of incubation time in both the HLM and MLM systems, thus suggesting a higher metabolic rate of THC, as opposed to CBD, in the liver. It was shown that the species difference in the metabolism of THC played a crucial role in the preclinical study of phytocannabinoids.

A key psychoactive metabolite of THC, 11-hydroxy-Delta(9)-Tetrahydrocannabinol, was previously identified as coming into effect after smoking marijuana, and its pharmacological activity turned out to be higher than THC [11]. In this paper, we found that it was the T8 metabolite. The T8 metabolite could further oxidate 11-COOH-THC (which was found to be T26 in our study) and combine easily with glucuronide to form a phase II metabolite, which may contribute to detoxification [12]. In a previous study, aldehydes, which are toxic products in the body, were able to be detoxified from the body due to their conjugation with glycine in vivo, which contributed to the aldehydes’ excretion from the body [13]. In addition, our study reported the formation of GSH and Cys conjugates of THC and CBD, when wither GSH or Cys was present. Notably, four metabolites, which were formed as a result of THC conjugating with GSH, were first found in 2018, and they contained 11-OH-THC-GSH, 11-COOH-THC-GSH, and two other unidentified metabolites [14]. Such findings may underlie some of the side effects of THC administration, including both the neuroactive properties and the cytotoxic effects. Although GSH conjugating with drug metabolites might be a protective mechanism that cells employ in order to neutralize the production of reactive intermediates, the excessive consumption of GSH during this process can lead to the depletion of GSH, which thus makes cells less able to cope with oxidative stress [15]. Cys is the synthetic precursor of GSH, meaning that Cys-conjugated THC metabolites may reduce the cellular pool of Cys, and thus further exacerbate oxidative stress. This might be linked to the cytotoxic effect of THC administration, which is the result of the depletion of reactive molecule scavengers. On the other hand, our results showed that the GSH and Cys conjugates of THC metabolites can readily bind to the CB1 receptor, which may contribute to the addictive property of THC, whereas CBD conjugates with GSH need to oxidate with CBD-hydroxyquinone [16]; this structure could increase steric hindrance when binding with the CB1 receptor. Cys itself is a ligand for the CB1 receptor, and it has been considered as a potentially excitatory toxin that can cause neurotoxicity in excess amounts [17]. Its conjugation with THC could cause THC metabolites to have an enhanced affinity with CB1, which is supported by the results from the simulated docking experiment in the present study. Such findings are also in line with previous findings concerning the hallucinogenic agents found in spice, myristicin and elemicin, which could conjugate with Cys [4,5]; therefore, the formation of the GSH- and Cys-conjugated products of THC metabolites may also be associated with the psychoactive and addictive effect of THC administration.

CYP450s are a type of drug-metabolizing enzyme, which may lead to adverse effects that enhance the toxicity of metabolites. In this paper, we used cDNA-expressed P450 enzymes and inhibitors to assess the role of specific enzymes in THC and CBD metabolite formation. During the formation of phase I metabolites and conjugated metabolites, THC metabolites were mainly modulated by CYP2C9 and CYP3A4 [18], whereas CBD metabolites were majorly modulated by CYP2C19 and CYP3A4 [19]. The GSH- and Cys-conjugated products of THC metabolites were majorly modulated by CY3A4 and CYP1B1, which are associated with the human pregnancy X receptor (PXR), the constitutive androstane receptor (CAR), and the aryl hydrocarbon receptor (AhR). PXR and CAR are associated with pharmacokinetics, the activation of which occurs through dependent CYP3A4 pathways that modulate drug metabolism [20], and AhR may control the expression of extrahepatic CYP1B1, and thus, xenobiotic toxicity and carcinogenesis [21]. 

The CB1 receptor is a member of the G protein-coupled receptor (GPCR) family, and is mainly distributed in the presynaptic nerve terminals in the central and peripheral nervous systems [22]. In marijuana users, the activation of CB1 triggers cannabinoid-induced central nervous system (CNS) effects, such as alterations in mood and cognition function [23]. It has been largely accepted that the binding of THC with the CB1 receptor is the main reason for its addictive properties. In accordance with a previous report [24], molecular docking was carried out using Discovery studio software in the present study, and the results showed that CBD had a low binding affinity with the CB1 receptor at the orthostatic binding sites, as opposed to THC, which had a high binding affinity with the receptor. These results were consistent with previous studies. THC binding with the CB1 receptor was more effective than CBD binding with the receptor because of steric hindrance in the structure of CBD [25]. In addition, we identified seven THC metabolites with higher CDOCKER INTERACTION energy and lower binding energy than the THC parent compound. All of these metabolites were produced by oxidation and di-oxidation during phase I of metabolism and conjugated metabolism. Notably, products formed as a result of THC conjugating with GSH and Cys metabolites displayed the highest binding affinity with CB1 receptors among all the THC metabolites, thus suggesting that these conjugated metabolites are the fundamental dominating mediators of the addictive property of THC. Nonetheless, downstream CB1 activation was affected by THC, and related metabolites and the manner in which they might be linked to neurotoxicity requires further examination. 

## 4. Materials and Methods

### 4.1. Chemicals and Reagents

THC and CBD were obtained from Sigma-Aldrich (Merck, Germany). Alpha-naphthoflavone, ticlopidine, ketoconazole, sulfaphenazole, methoxsalen, trimethoprim, quindine, nicotinamide adenine dinucleotide phosphate (NADPH), ethyl acetate, GSH, Cys, and formic acid were purchased from Sigma-Aldrich (St. Louis, MO, USA). The MLM and HLM systems were provided by Bioreclamationivt Inc. (Hicksville, NY, USA). Recombinant human P450 enzymes were purchased from Xenotecch, LLC (Kansas City, MO, USA). All organic reagents were of the highest commercially available grade.

### 4.2. Metabolism of THC and CBD in the MLM and HLM Systems

In vitro metabolism of THC and CBD was conducted in a 96 wells plate, and each incubation was conducted in triplicate [26]. After a series of pre-experimental tests, the final concentrations of GSH and CBD, at 0.2, 1, 5, and 25 μM, were tested with HLM co-incubation. The incubation system (180 μL) contained 25 μM THC or 25 μM CBD, and the final concentration of the HLM or MLM was placed in a 0.5 mg protein/mL phosphate-buffered saline solution (PBS, pH = 7.4). After pre-incubation for 5 min at 37 °C, with shaking at 400 rpm, this system was fortified with 20 μL of 10 mM NADPH, and it continued for 40 min at 37 °C, with shaking at 400 rpm in a MicBio Ⅱ incubator (Abson, USA). Reactions were terminated with the addition of 200 μL of ice-cold acetonitrile. The mixture was vortexed for 60 s, followed by centrifugation at 16,200× *g* for 20 min at 4 °C. Two control groups were pre-prepared using the same protocol, although the incubation system was not treated with NADPH or drugs, respectively. A 5 μL aliquot of the supernatant was collected for Thermo Scientific™ Vanquish™ flex ultra-high-performance liquid chromatography Q Exactive^TM^ MS analysis (UHPLC Q-Exactive).

### 4.3. Trapping Reactive Metabolites using GSH and Cys 

To investigate the potential of THC and CBD when forming electrophilic metabolites in the presence of nucleophiles, either 400 μM GSH or 400 μM Cys was added to MLM/HLM treated with THC or CBD, as described in the previous section [5]. Five μL of supernatants were collected for UHPLC-Q-Exactive MS analysis.

### 4.4. Metabolism of THC and CBD in Human Recombinant CYP450s

In order to understand the contribution of different CYPs in the metabolism of THC and CBD, a 25 μM substrate (THC or CBD) was mixed with 2 pmol/mL of each of the cDNA-expressed P450 enzymes (control, CYP1A1, CYP1A2, CYP1B1, CYP2A6, CYP2B6, CYP2C19, CYP2C8, CYP2C9, CYP2D6, CYP2E1, CYP3A4, CYP3A5, CYP4A11) to comprise a final volume of 180 μL. The reaction was initiated with the addition of 20 μL NADPH (10 mM) and it stopped after 40 min with the addition of 200 μL of ice-cold acetonitrile. The solution was centrifuged at 16,200× *g* for 20 min at 4 °C, and a 5 μL aliquot of the supernatant was collected for UHPLC Q-Exactive MS analysis. 

To investigate the contribution of CYPs, and to identify the CYPs responsible for the formation of the GSH- or Cys-conjugated products of THC, inhibitor assays were carried out in a THC-treated MLM system in the presence of 400 μM GSH/Cys. The final concentrations of each chemical inhibitor, and the corresponding CYP target, were shown as follows [5]: α-naphthoflavone (1.0 μM; CYP1A1/2 inhibitor), ketoconazole (100 μM; CYP3A4 inhibitor), ticlopidine (100 μM; CYP2C19 and CYP2B6 inhibitor), methoxsalen (20 μM; CYP2A6/13 inhibitor), trimethoprim (2.5 μM; CYP2C8 inhibitor), sulfaphenazole (100 μM; CYP2C9 inhibitor), quinidine (5.0 μM; CYP2D6 inhibitor), and resveratrol (10 μM; CYP1B1 inhibitor). The control group was carried out in the same vehicle, but in the absence of inhibitors. The reaction mixtures were collected for UHPLC Q-Exactive MS analysis to determine whether THC conjugated with GSH/Cys.

### 4.5. UHPLC Q-Exactive MS Analysis

The analysis of the microsomes was conducted using the UHPLC Q-Exactive (Thermo Fisher Scientific, San Jose, CA, USA) system, and analyte separations were achieved using a HSS T3–C18 column (2.1 mm × 100 mm, 1.8 μm) at 25 °C. The analytes were eluted with a gradient elution of 0.1% formic acid in water (A) and acetonitrile (B). The gradient procedure was performed as follows: 1% B for 0–1 min, 1–70% B for 1–3 min, 70–100% B for 3–15 min. 100–1% B for 15–18 min. Other conditions were set as follows: flow rate: 0.3 mL/min; capillary temperature: 350 °C; gas flow: 15 L/min; sheath gas flow rate: 35 L/min; capillary voltage: 3.7 kV; injection volume: 5 μL. The mass spectrometer was operated in a positive ionization mode. The MS/MS fragment information was detected using the following MS/MS conditions: the collision energy was set at 15, 25 and 35 eV, respectively; the orbitrap resolution was set to 70,000; Xcalibur software (Version 4.3, Thermo Fisher Scientific, San Jose, CA, USA) was used to control the LC-HRMS system and for data acquisition [26].

### 4.6. Data Processing, Feature Selection, and Metabolite Annotation

Thermo Xcalibur Qual Browser software (Thermo Fisher Scientific, San Jose, CA, USA) was used to detect THC and CBD metabolites. Chromatographic analysis and data processing was performed using MS-DIAL software. The parameters were set as follows: retention time range = 0–15 min, minimum peak height = 10,000, and the parent ions of [M + H]^+^ were selected for screening metabolites when the software was set to the positive mode. The processed data matrix was exported as an excel file containing the peak number, retention time, *m/z*, and integrated peak area. Unsupervised principal analysis (PCA) was applied to select discriminatory features (SIMCA v.14.1 software (Kinnelon, NJ, USA). The selected features were searched against the MS/MS analysis to identify the THC and CBD metabolites. Finally, the structures of these tentatively assigned features were analyzed and confirmed using MS/MS fragmentation patterns.

The structures of THC and CBD metabolites were drawn using ChemBioDraw Ultra v.13.0 software (Cambridge Soft Corporation, Cambridge, MA, USA), and the Clog P values of THC and CBD metabolites were calculated using the chemical properties window. Clog P is a potential indicator that reflects the polarity of a compound. A compound with a low Clog P value exhibits high polarity, which corresponds to an early elution time in reversed-phase chromatography [27]. The isomeric metabolites of THC and CBD can therefore be distinguished from one another through the use of the retention time and Clog P value.

### 4.7. Molecular Docking Prediction Concerning How the CB1 Receptor Interacts with Metabolites

To further understand the relationship between the metabolism of THC and drug addiction, a molecular docking simulation was utilized to understand the mechanism of interaction between the CB1 receptor and THC and CBD metabolites. The crystalline structure of the human CB1 receptor was confirmed in a previous study, which also depicted a model of the CB1 active site based on X-ray diffraction [28]. Molecular docking was simulated using the Discovery Studio 2020 in order to visually characterize the affinity between metabolites and the CB1 receptor. The standard three-dimensional structure of metabolites (*mol) as ligands was established by Chemdraw 3D v.19.0 software (Cambridge, MA, USA). The receptor structure of CB1 (PDB ID:5U09) was downloaded from the RCSB Protein Data Bank (https://www.rcsb.org/) (accessed on 14 October 2021) and imported into the Discovery Studio 2020 (Accelrys Software Inc., San Diego, CA, USA). The structure of the CB1 receptor was modified by removing water and adding hydrogen. The partially flexible CDOCKER program was used to determine special binding sites and the receptor radius. The molecular docking results were analyzed to determine the -CDOCKER energy score, -CDOCKER INTERATION energy, and binding energy.

### 4.8. Statistical Analysis

Experimental data were presented as a mean ± SD. Statistical analysis of the two-group comparison was performed using a Student’s *t*-test in GraphPad Prism 8.0. software. Data normality was confirmed using the Shapiro–Wilk test prior to conducting the *t*-test. When repeating the two-group comparison between the control group and experimental group, the statistical significance was determined using the Student’s *t*-test with multiple testing correction. *P* < 0.05 was considered to be statistically significant.

## 5. Conclusions

In summary, the present study has comprehensively measured and compared the metabolic intermediates and products of the metabolism of THC and CBD using in vitro systems. A total number of 59 drug metabolites were identified using LC–MS, including 19 previously undescribed metabolites. The metabolic rate of THC in the liver microsome was found to be higher than that of CBD, and four conjugates (two THC-GSH and two THC-Cys) were reported as products that were caused by the metabolism of THC. Molecular docking has confirmed that THC-GSH and THC-Cys can readily bind to the CB1 receptor, with a higher affinity and greater stability than THC, which may be linked to, or may mediate, some of the THC-induced neuroactive effects. Lastly, we reported that CYP3A4 and CYP1B1 are the key enzymes responsible for the formation of the THC-GSH and THC-Cys conjugates. Further studies are needed to illustrate the exact role of the metabolism of THC and the cellular mechanism underlying its neurotoxic and psychoactive effects following cannabis exposure.

## Figures and Tables

**Table 1:** Summary of THC metabolites produced with metabolisms in vitro.

Number	RT (min)	Clog P	Observed *m/z* [M + H]	Delta ppm	MS/MS Fragments	Predicted Molecular Formula	Identification	Source	NAPDH Independent
T0	10.32	7.24	315.2308	−4.98	93, 193, 259, 297, 315	C_21_H_30_O_2_	THC	HLM, MLM	-
*T1	9.89	6.95	313.2161	−1.94	95, 175, 193, 257, 271, 313	C_21_H_28_O_2_	THC-2H	HLM, MLM	-
*T2	10.2	7.16	313.2163	−1.24	95, 193, 217, 271, 295, 313	C_21_H_28_O_2_	THC-2H	HLM, MLM	-
*T3	4.65	6.51	311.2009	−0.64	95, 185, 213, 255, 269, 311	C_21_H_26_O_2_	THC-4H	HLM, MLM	-
*T4	4.83	6.65	311.1996	−4.88	95, 191, 255, 269, 293, 311	C_21_H_26_O_2_	THC-4H	HLM, MLM	NADPH
*T5	5.35	6.86	311.1997	−4.56	93, 217, 241, 269, 296, 311	C_21_H_26_O_2_	THC-4H	HLM, MLM	NADPH
*T6	6.68	-	311.1996	−4.98	93, 193, 217, 223, 265, 311	C_21_H_26_O_2_	THC-4H	HLM, MLM	NAPDH
T7	9.48	7.39	311.2000	−3.53	195, 223, 293, 311	C_21_H_26_O_2_	THC-4H	HLM, MLM	-
T8	6.81	5.35	331.2261	−3.50	95, 193, 257, 271, 313	C_21_H_30_O_3_	THC+O	HLM, MLM	NAPDH
T9	8.38	5.45	331.2261	−3.62	95, 193, 231, 273, 313	C_21_H_30_O_3_	THC+O	HLM, MLM	NAPDH
T10	4.51	-	329.2115	−0.72	193, 217, 245, 301, 329	C_21_H_28_O_3_	THC+O-2H	HLM, MLM	NAPDH
T11	4.64	5.27	329.2100	−5.10	213, 261, 311, 329	C_21_H_28_O_3_	THC+O-2H	HLM, MLM	-
T12	4.81	5.3	329.2107	−3.03	95, 191, 255, 311, 329	C_21_H_28_O_3_	THC+O-2H	HLM, MLM	NAPDH
T13	4.98	5.49	329.2100	−4.92	95, 191, 255, 311, 329	C_21_H_28_O_3_	THC+O-2H	HLM	NAPDH
T14	5.09	5.64	329.2114	−1.03	95, 191, 213, 255, 311, 329	C_21_H_28_O_3_	THC+O-2H	HLM	NAPDH
T15	7.47	6.16	329.2100	−4.92	217, 231, 245, 287, 311, 329	C_21_H_28_O_3_	THC+O-2H	MLM	NAPDH
T16	5.50	3.64	349.2370	−2.57	193, 259, 313, 349	C_21_H_32_O_4_	THC+2H+2O	MLM	NAPDH
T17	5.35	3.59	347.2216	−1.84	191, 273, 311, 329, 347	C_21_H_30_O_4_	THC+2O	HLM	NAPDH
T18	5.4	-	347.2215	−2.10	191, 273, 311, 329, 347	C_21_H_30_O_4_	THC+2O	MLM	NAPDH
T19	6.00	4.42	347.2216	−1.75	193, 206, 233, 273, 311, 329, 347	C_21_H_30_O_4_	THC+2O	MLM	NAPDH
T20	6.39	5.73	333.2414	−4.80	217, 217, 261, 289, 315, 333	C_21_H_32_O_3_	THC+O+2H	MLM	-
T21	4.06	4.17	345.2053	−3.88	191, 233, 243, 285, 345	C_21_H_28_O_4_	THC+2O-2H	MLM	NAPDH
T22	4.48	4.41	345.2052	−3.96	191, 261, 285, 309, 327, 345	C_21_H_28_O_4_	THC+2O-2H	HLM, MLM	NAPDH
T23	5.06	5.04	345.2053	−3.79	229, 271, 285, 327, 345	C_21_H_28_O_4_	THC+2O-2H	HLM, MLM	NAPDH
T24	4.72	4.83	345.2053	−3.79	253, 271, 327, 345	C_21_H_28_O_4_	THC+2O-2H	MLM	-
T25	4.31	4.27	345.2053	−3.70	253, 261, 267, 309, 345	C_21_H_28_O_4_	THC+2O-2H	HLM, MLM	NAPDH
T26	5.23	5.7	345.2054	−3.44	229, 271, 285, 327, 345	C_21_H_28_O_4_	THC+COOH	HLM, MLM	NAPDH
T27	4.97	5.29	345.2054	−3.62	95, 207, 271, 309, 327, 345	C_21_H_28_O_4_	THC+COOH	HLM, MLM	NAPDH
T28	4.58	-	345.2058	−2.31	253, 271, 327, 345	C_21_H_28_O_4_	THC+2O-2H	HLM	NAPDH
*T29	4.53	3.96	618.2827	−3.39	129, 199, 311, 618	C_31_H_43_N_3_O_8_S	THC+GSH-2H	HLM/ MLM+ GSH	-
T30	4.48	2.18	634.2780	−2.83	327, 359, 487, 616, 634	C_31_H_43_N_3_O_9_S	THC+GSH+O-2H	HLM/ MLM+ GSH	-
*T31	4.87	4.65	432.2223	3.47	199, 297, 311, 432	C_24_H_33_NO_4_S	THC+CYS-2H	HLM/ MLM+ Cys	-
*T32	4.83	2.86	448.2141	−3.57	245, 327, 399, 448	C_24_H_33_NO_5_S	THC+Cys+O-2H	HLM/ MLM+ Cys	-

* Indicates novel metabolites found in this study. T0, THC; +O, hydroxylation; −2H, desaturation; +COOH, carboxylic acid; GSH, glutathione; Cys, Cysteine.

**Table 2:** The contribution of CYPs during the formation of THC metabolites (The total of each metabolite was 100%).

	CYP1A1	CYP1A2	CYP1B1	CYP2A6	CYP2B6	CYP2C19	CYP2C8	CYP2C9	CYP2D6	CYP2E1	CYP3A4	CYP3A5	CYP4A11
T1	-	-	-	10.41	11.16	-	-	-	-	7.99	27.39	36.62	6.44
T2	-	7.35	9.29	13.48	12.39	-	9.60	-	7.91	10.50	14.20	7.58	7.70
T3	-	-	-	-	-	-	-	-	-	-	-	100.00	-
T4	-	-	-	-	-	52.58	-	-	43.90	-	3.52	-	-
T5	-	3.99	-	-	-	81.65	-	-	-	-	14.36	-	-
T6	-	24.06	-	26.89	-	-	-	-	-	-	36.22	12.83	-
T7	4.77	5.62	6.91	9.76	11.93	3.00	9.24	7.40	7.06	11.17	8.89	8.19	6.06
T8	2.76	0.08	0.18	-	0.79	27.12	4.12	61.98	2.42	-	0.26	0.27	0.02
T9	-	1.20	1.22	1.35	1.41	-	1.25	-	-	1.19	54.52	36.74	1.13
T11	23.87	-	-	-	-	-	2.83	-	6.39	-	7.87	59.04	-
T12	-	3.82	-	-	-	-	7.49	-	65.29	-	23.40	-	-
T13	-	-	-	-	-	-	-	-	-	-	100.00	-	-
T14	93.09	-	-	-	-	-	-	-	3.55	-	3.36	-	-
T15	6.70	7.71	8.02	8.93	9.20	7.29	8.74	8.30	6.79	8.00	7.31	6.36	6.66
T16	7.32	8.80	9.61	10.62	10.61	6.61	10.49	8.79	6.26	8.36	12.55	-	-
T17	-	3.62	0.88	-	-	8.29	-	-	-	-	28.31	58.90	-
T18	10.34	-	-	-	-	84.09	-	-	-	-	5.57	-	-
T19	3.81	4.61	4.19	4.76	5.79	3.85	4.89	4.60	3.01	3.94	15.44	37.72	3.39
T20	3.65	8.23	9.72	5.45	8.52	6.69	9.82	10.85	2.36	4.25	6.66	13.35	10.45
T22	29.04	1.54	8.11	-	-	24.44	2.88	-	23.59	-	2.96	7.43	-
T23	39.72	3.87	0.92	-	-	36.47	-	0.59	-	-	18.43	-	-
T24	30.16	2.04	-	-	-	27.04	8.41	0.84	-	-	9.22	22.28	-
T25	-	-	5.56	-	-	-	-	-	-	-	20.83	73.61	-
T26	-	-	0.87	-	-	54.76	10.43	0.57	-	-	13.84	19.55	-
T27	25.62	0.62	1.58	-	-	10.81	3.88	0.45	-	-	12.84	44.20	-
T28	-	1.87	4.78	-	-	22.19	6.52	-	59.93	-	4.70	-	-

c-DNA-expressed CYPs (Control, CYP1A1, CYP1A2, CYP2B6, CYP2C19, CYP2C8, CYP2C9, CYP2D6, CYP2E1, CYP3A4, CYP3A5, CYP4A11) were used to detect the roles of individual CYPs in the metabolism of THC. All samples were analyzed using UHPLC Q-Exactive MS. All data are expressed as a mean (*n* = 3). The numbers in the table are percentages, and they represent the percentage of the total of each metabolite that was formed across all cytochromes, of the P450 enzymes tested.

## Data Availability

Not applicable.

## Supplementary Materials

The following supporting information can be downloaded at: https://www.mdpi.com/article/10.3390/molecules27217573/s1. Figure S1: The MS/MS of THC and CBD. Figures S2 and S3: Identification of THC and CBD typical metabolites in MS/MS, Figure S4: In vitro metabolism of THC and CBD, Figure S5: Molecular interaction of THC and CBD with CB1 receptor, Figure S6: Proposed metabolic pathways of CBD.

## Sample Availability

Samples of the compounds are available from the authors.
