# Supplementary material for: Comparative Metabolomic Profiling of the Metabolic Differences of Δ9-Tetrahydrocannabinol and Cannabidiol
Source: Molecules. 2022 Nov 4;27(21):7573. doi: 10.3390/molecules27217573 (PMC9657426; doi:10.3390/molecules27217573)
Supplement: Supplementary file 1 [file molecules-27-07573-s001.zip › molecules-1942669-supplementary.pdf]

Supplementary Materials

Comparative metabolomic profiling of the metabolic differences between Δ9-tetrahydrocannabinol and cannabidiol

Qianru Rao [1,2 †], Ting Zhang [1,3 †], Manyun Dai [1,3], Bin Li [2], Qianlun Pu [4], Min Zhao [1], Yan Cheng [1], Dongmei Yan [2], Qi Zhao [1], Zhanxuan E.Wu [1], Fei Li[1]

[1] Laboratory of Metabolomics and Drug-Induced Liver Injury, Frontiers Science Center for Disease-Related Molecular Network, West China Hospital, Sichuan University, Chengdu 610041, China

[2] Academician Workstation, Jiangxi University of Chinese Medicine, Nanchang 330004, China

[3] States Key Laboratory of Phytochemistry and Plant Resources in West China, Kunming Institute of Botany, Chinese Academy of Sciences, Kunming 650201, China

[4] Advanced Mass Spectrometry Center, Research Core Facility, Frontiers Science Center for Disease-Related Molecular Network, West China Hospital, Sichuan University, Chengdu 610041, China.

[†] These authors contributed equally to this work.

1. Identification of THC and CBD metabolites

1.1 Desaturation metabolites

Desaturation metabolites of THC (T1–T7) and CBD (C1–C7) have not been previously reported, a fact which was identified in the main manuscript (the last paragraph of section 3.2). Desaturation metabolites of THC and CBD, concerning MS/MS spectrums and fragmentation patterns, were separately shown in Figures S1 and 2.

1.2 Oxidative metabolites

The chemical formula of metabolites T8 and T9 was $C_{21}H_{28}O_2$, according to the observed $[M+H]^+$ at *m/z* 331.2271^+ and 331.2264^+. T8 and T9 are isomers, and they were eluted successively at 6.81 and 8.38 min. The diagnostic product, which was an ion at *m/z* 313, was observed as a result of the loss of 18 Da, thus suggesting that a molecular H_2O was lost from a protonated molecular ion. The positions of the oxidation reaction usually occurred at the methylcyclohexene ring. According to the fragment interpretation, the elemental formula was assigned as $C_{21}H_{26}O_2$, as a result of the oxidation of the methylcyclohexene ring. T8 had other characteristic fragment ions, 193, 257, 271, and 313, which were formed by the individual or combined losses of C_3H_6, CH_2, OC_3H_6, or H_2O. According to the Clog P value, it was speculated that the position of hydroxylation was at C8 in T8, and the Clog P value of T8 was 5.35. The position of hydroxylation was at C11 in T9, and the Clog P value of T9 was 5.45. Metabolites T17–T19 were observed in the extracted chromatogram at *m/z* 347.22156^+, 347.22147^+, and 347.22159^+; these values were 32 Da (2O) higher than that of the THC parent compound, thus indicating that these metabolites were the dihydroxylated products of THC. T17 had other characteristic fragment ions, 191, 273, 311, and 329, which were formed by the individual or combined losses of C_5H_{10}, C_3H_6, or H_2O. T17 and T18 had the typical fragment ion *m/z* 191($[M+H]^+$), and T19 had the typical fragment ion 193($[M+H]^+$), thus indicating that the dihydroxylated position in T19 might be located in the pentyl side chain, but the dihydroxylated position in T17 and T18 might be located in the THC ring; therefore, we may speculate that, with regard to the structure of T17, the position of dihydroxylation may be located at C8 and C1’. The dihydroxylated position of T18 might be located at C11 and C1’.

Similarly, the monohydroxylated and dihydroxylated metabolites of CBD were also observed. The chemical formula of metabolites C8–C11 was $C_{21}H_{30}O_3$, according to the $[M+H]^+$, at *m/z* 331.2262^+,

331.2276$^+$, 331.2263$^+$, and 331.2258$^+$; these values were 16 Da(O) higher than that of the CBD parent compound, thus indicating that these metabolites were the mono-hydroxylated products of CBD. The retention times of C8–C11 were 5.46, 5.75, 7.04, and 7.45 min. According to the Clog P values of C8 to C11, the positions of hydroxylation might be located in either the 2”, 6, 7, or 1”-hydroxyl cannabidiol (C8–C11), respectively. The chemical formula for C18 and C19 was $C_{21}H_{30}O_4$, according to *m/z* 347.2216$^+$ and 347.2209$^+$ ([M + H]$^+$); these values were 32 Da(2O) higher than that of the CBD parent compound, thus indicating that these metabolites were the dihydroxylated products of CBD, and they were identified as 5,6-dihydroxy-cannibidiol (C18) and 1”,3”-dihydroxy-cannibidiol (C19).

1.3 Hydroxylated with desaturated metabolites

T10 and T11, and C12 to C16, were subjected to oxidation and a desaturation reaction. Their chemical formula was $C_{21}H_{28}O_3$, according to the [M + H] $^+$ at *m/z* 329.2100$^+$ to 329.2114$^+$; these values were 14 Da (O-2H) higher than that of the THC and CBD parent compounds. The retention times of T10 and T11 were 4.51 and 4.64 min. The metabolites T24, T25, and T28, and C21 and C22, were observed in the extracted chromatogram at *m/z* 345.2053$^+$, 345.2053$^+$, 345.2058$^+$, 345.2053$^+$, and 345.2077$^+$ ([M+H] $^+$); these values were 30 Da (2O-2H) higher than those of the THC and CBD parent compounds. The loss of H_2O (18 Da, *m/z* 327$^+$) was observed in the MS/MS analysis, which suggested that T24, T25, and T28, and C2 and C22, were the deoxidated, desaturated metabolites of THC and CBD.

1.4 Oxidated and desaturated metabolites

The T20 metabolites were eluted at 6.39 min, with an observed molecular ion at *m/z* 333.2414 ([M + H]$^+$); this value was 18 Da higher than the quasi-molecular ion of oxidation. The MS/MS spectrum displayed typical fragment ions at *m/z* 289$^+$, which corresponded with the successive loss of OC_2H_4 (44 Da), thus suggesting that oxidation occurred at the 5’-position and reduction could occur at the ring of ethylene position.

The T16 and C23 metabolites were generated as a result of dihydrolation and a reduction reaction. In the spectrum of T16, the characteristic fragment ions at *m/z* 313$^+$ were found by the successive loss of H_2O groups from the fragment ion at 349. According to the elemental formula of $C_{21}H_{32}O_4$, and the fragment interpretation, both metabolites were the result of oxidation at the C5’-position and C8-

position. Similarly, C23 metabolites may also have been produced as a result of the deoxidation–reduction reaction. The loss of H_2O (18 Da, *m/z* 331^+), CO (28 Da, *m/z* 303^+), and C_2H_4 (28 Da, *m/z* 275^+) were observed in the MS/MS analysis of C23.

1.5 Metabolites from other types of oxidation reaction

THC can be oxidated to aldehydes or ketone metabolites (T12–T15). T12–T15 were assigned the molecular formula, $C_{21}H_{28}O_3$, which correspond with the $[M + H]^+$ from *m/z* 329.2107^+ to 329.2113^+; these values were 14 Da (O-H_2) higher than the THC parent compound. The retention times of T12–T15 were 4.81, 4.98, 5.09, and 7.47 min, respectively. In accordance with the differences between retention times, the metabolites of the aldehyde and ketone positions were different. After further analysis of the Clog P value, the Clog P values of T12–T15 were 5.3, 5.49, 5.64, and 6.16, meaning that the C11-position could be aldehyde. The formation of ketone metabolites included T12, T13, and T15, the locations of which were speculated to be at the C8-position, C7-position, and C1'-position. T26 and T27 were the carboxylic acid metabolites of THC. THC conjugated with carboxylic acid at 11-COOH. The chemical formula of metabolites T26 and T27 was $C_{21}H_{28}O_4$, according to the $[M + H]^+$, at *m/z* 345.2054^+ and 345.2054^+; these values were 30 Da (2O-2H) higher than that of the THC parent compound. These metabolites were the carboxylic products of THC, and they were identified as being 11-carboxylic acid-tetrahydrocannabinol (T26) and 5'-carboxylic acid- tetrahydrocannabinol (T27); these values correspond with the values of other THC products.

2. Comparison of the THC and CBD metabolisms in HLM and MLM

In a HLM incubation system, the THC metabolites were dominated by T8, and the relative abundance of mono-hydroxylated products (T8 and T9) accounted for 54% of the THC metabolites (Figure S3A). With regard to CBD metabolites, the total percentage of mono-hydroxylated products (C8–C10) was 7%, the percentage of desaturated products (C1–C3) was 4%, the percentage of dihydroxylated products (C18) was 1%, and the percentage of mono-hydroxylated products that had been desaturated (C13–C15) was 3%, compared with the CBD parent compound, respectively (Figure

S3B).

In a MLM incubation system that used THC, the total percentage of mono-hydroxylated products (T8 and T9) was 358%, the percentage of oxidated products (T15) was 342%, the percentage of mono-hydroxylated products that had been dehydrogenated (T10 and T11) was 27%, the percentage of dihydroxylated products (T18 and T19) was 130%, and the percentage of oxidated products that had been hydroxylated (T21–T25) was 99%, compared with the THC parent compound (Figure S3C). As for CBD metabolites in a MLM system, the relative abundance of desaturated products and di-desaturation formations (C1 and C2, and C4 to C6) were divided by 14% and 6%, compared with the CBD parent compound. Indeed, mono-hydroxylated products (C8–C11) were divided by 31%, mono-hydroxylated products that had been dehydrogenated (C12–C16) were divided by 77%, and dihydroxylated products (C18–C19) were divided by 40%, compared with the CBD parent compound (Figure S3D).

The metabolic rate of THC was higher than CBD in the HLM system, which was 62% in THC, and 28% in CBD (Figure S3E), respectively. Similarly, the metabolic rate of THC (95%) was higher than CBD (80%) in the MLM system (Figure S3F). These results confirmed the different metabolic rates of THC and CBD in these microsomal incubation systems.

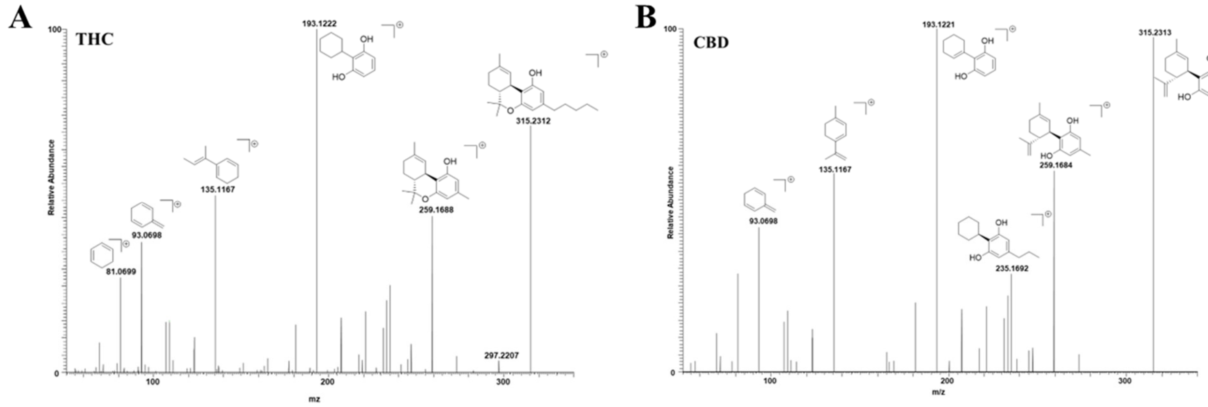

Figure S1. The MS/MS of THC (A) and CBD (B).

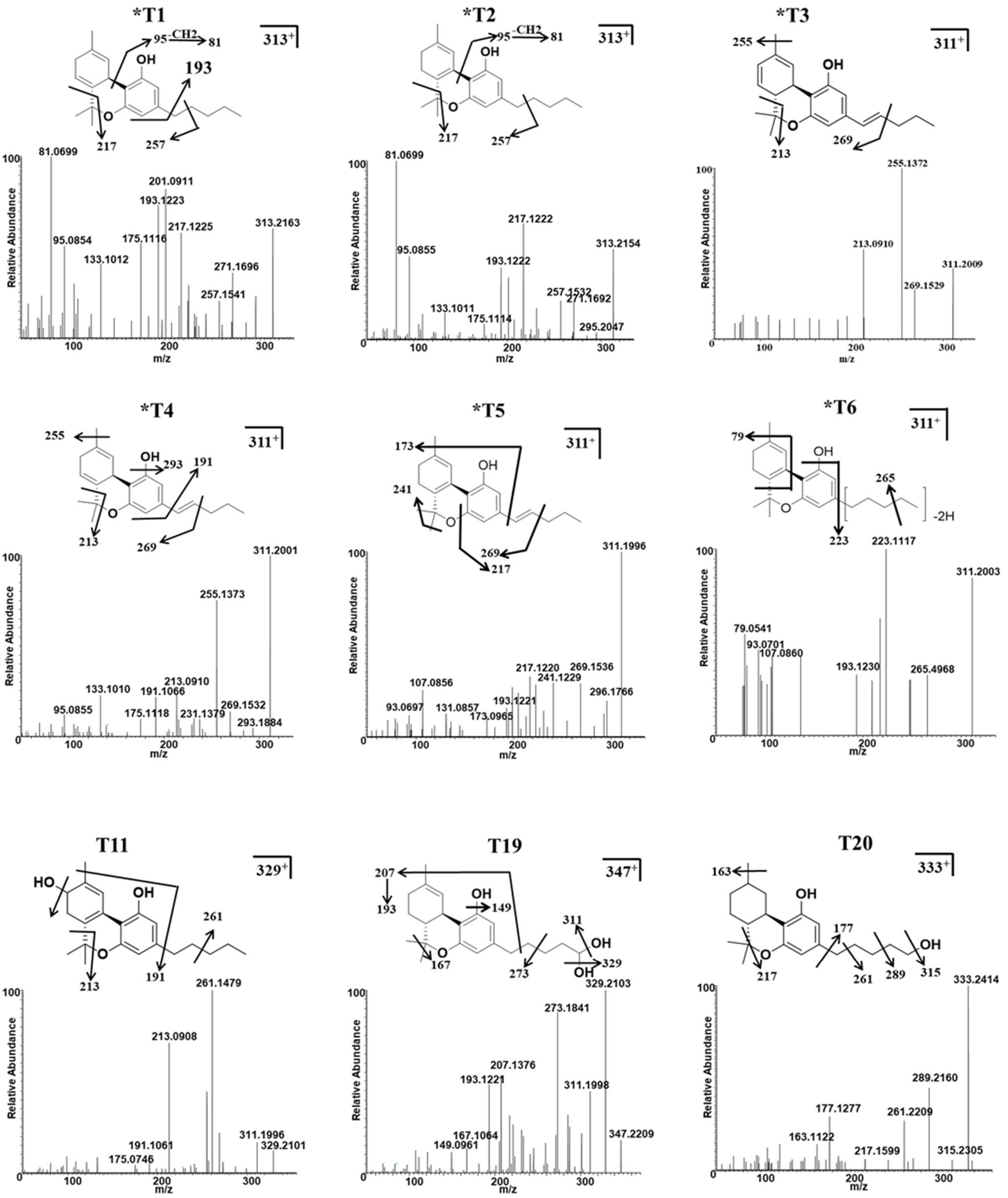

Figure S2. Identification of THC typical metabolites in MS/MS. * Represents undescribed metabolites found in this study.

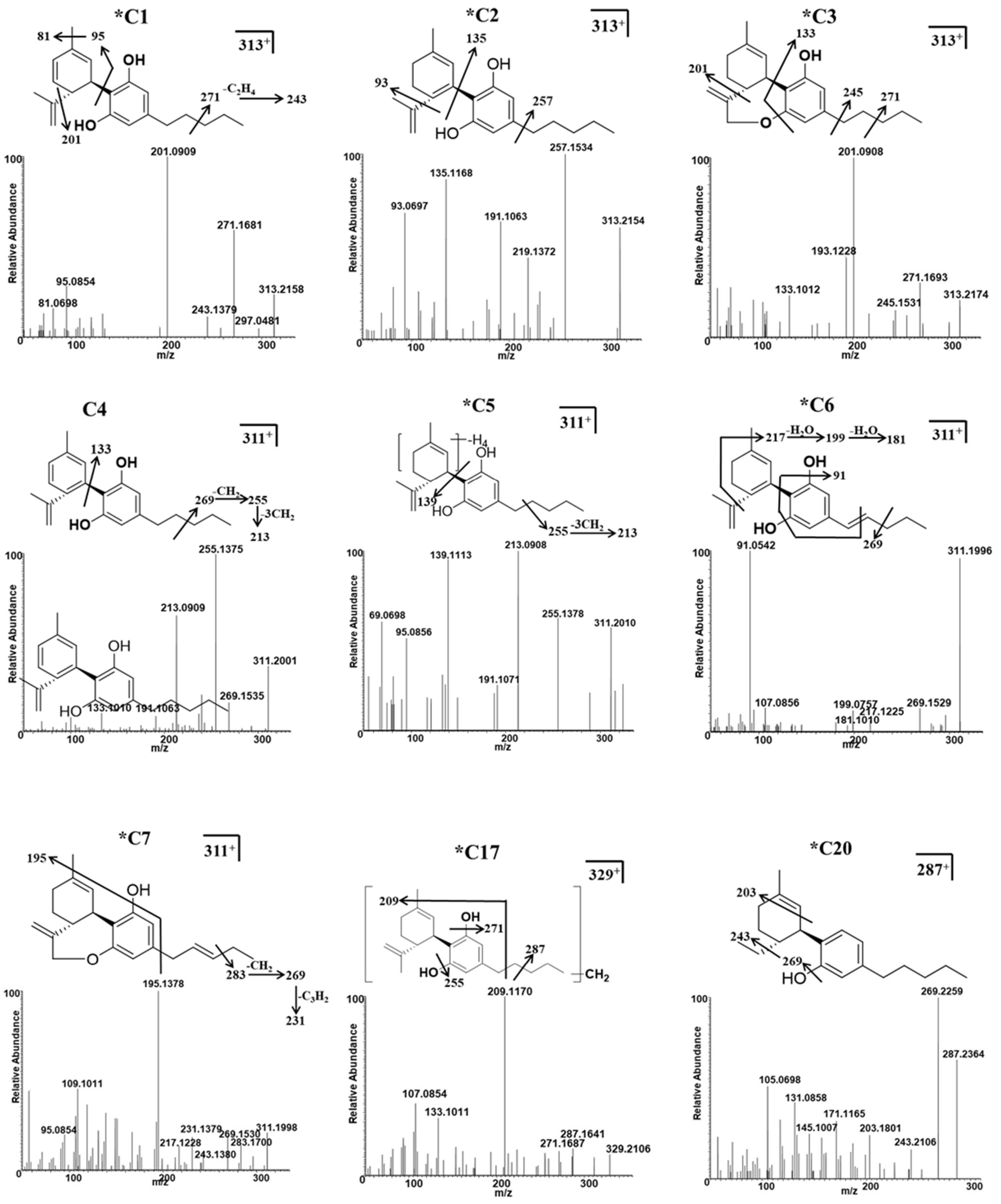

Figure S3. Identification of typical CBD metabolites in MS/MS. * Represents undescribed metabolites found in this study.

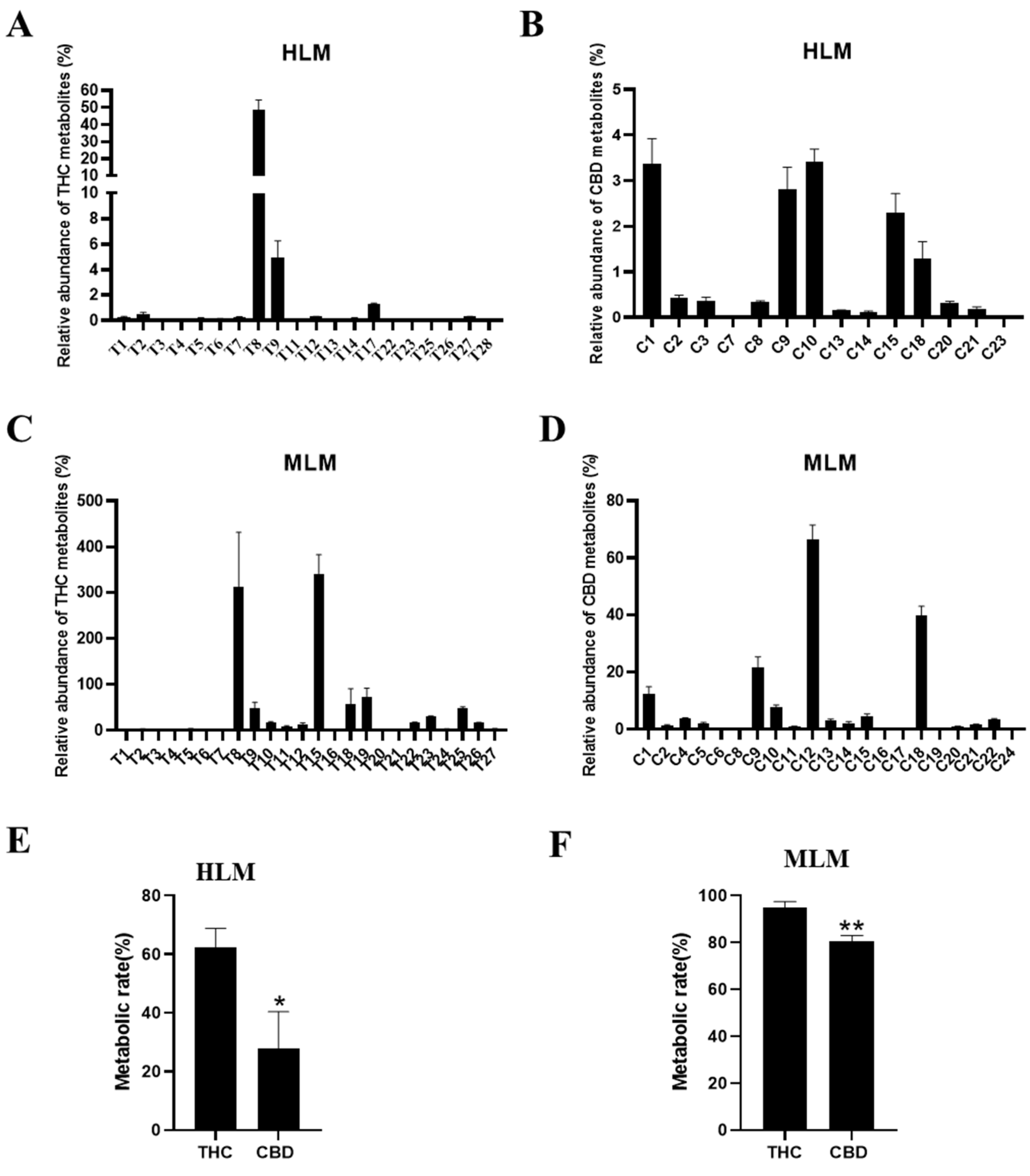

Figure S4. In vitro metabolisms of THC and CBD. (**A-B**) The relative abundance of THC metabolites in HLM/MLM. (**C-D**) The relative abundance of CBD metabolites in HLM/MLM. (**E-F**) Metabolic rate of THC and CBD in HLM/MLM. All samples were analyzed using UHPLC Q-Exactive MS. The prototypes of THC and CBD were integrated up to 100%. The data were represented as the means ± SD (n=3). Statistical analysis between the groups was conducted using the Student's independent *t*-test. *$P < 0.05$, ** $P < 0.01$, *** $P < 0.001$.

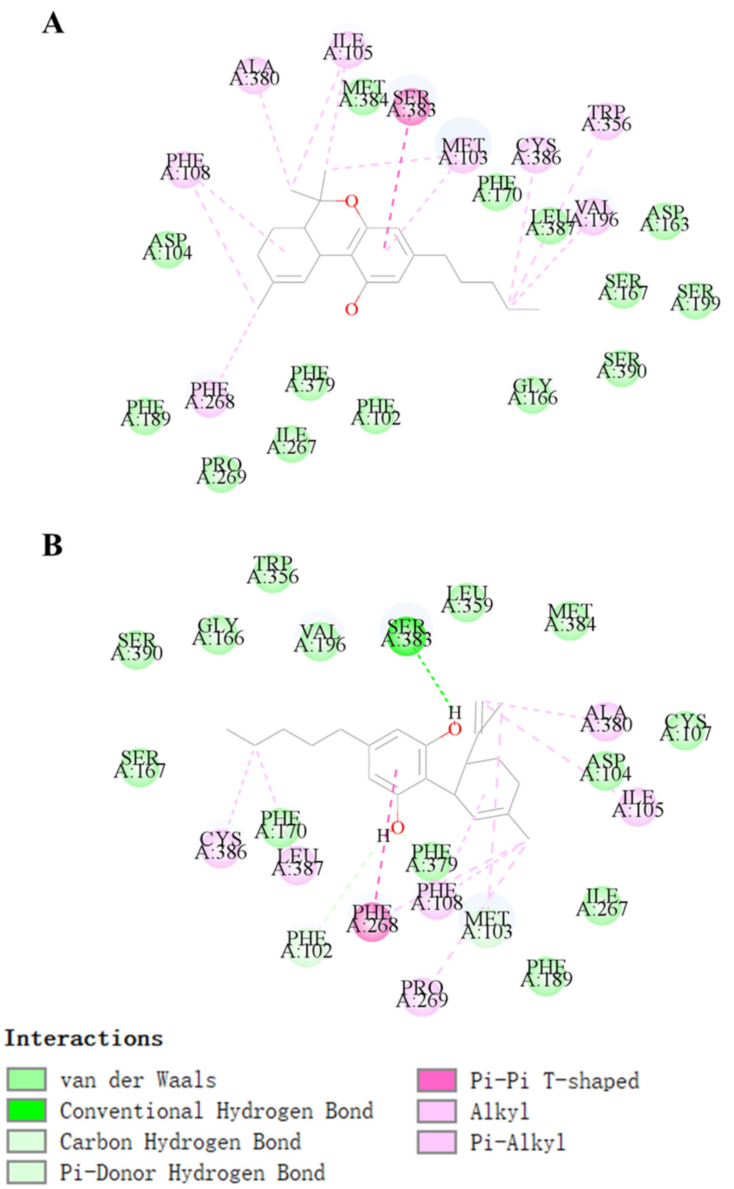

Figure S5. Molecular interaction between THC and CBD on the CB1 receptor. The lowest energy docked pose and 2D interaction map in the THC (**A**) and CBD (**B**) binding sites of the CB1 receptor. The green color and the red color represent the hydrogen bond and conjugated linkage interaction between the target and the ligand molecules, respectively. Active site residues involved in the interaction are represented in blue.

Figure S6. Proposed metabolic pathways of CBD.

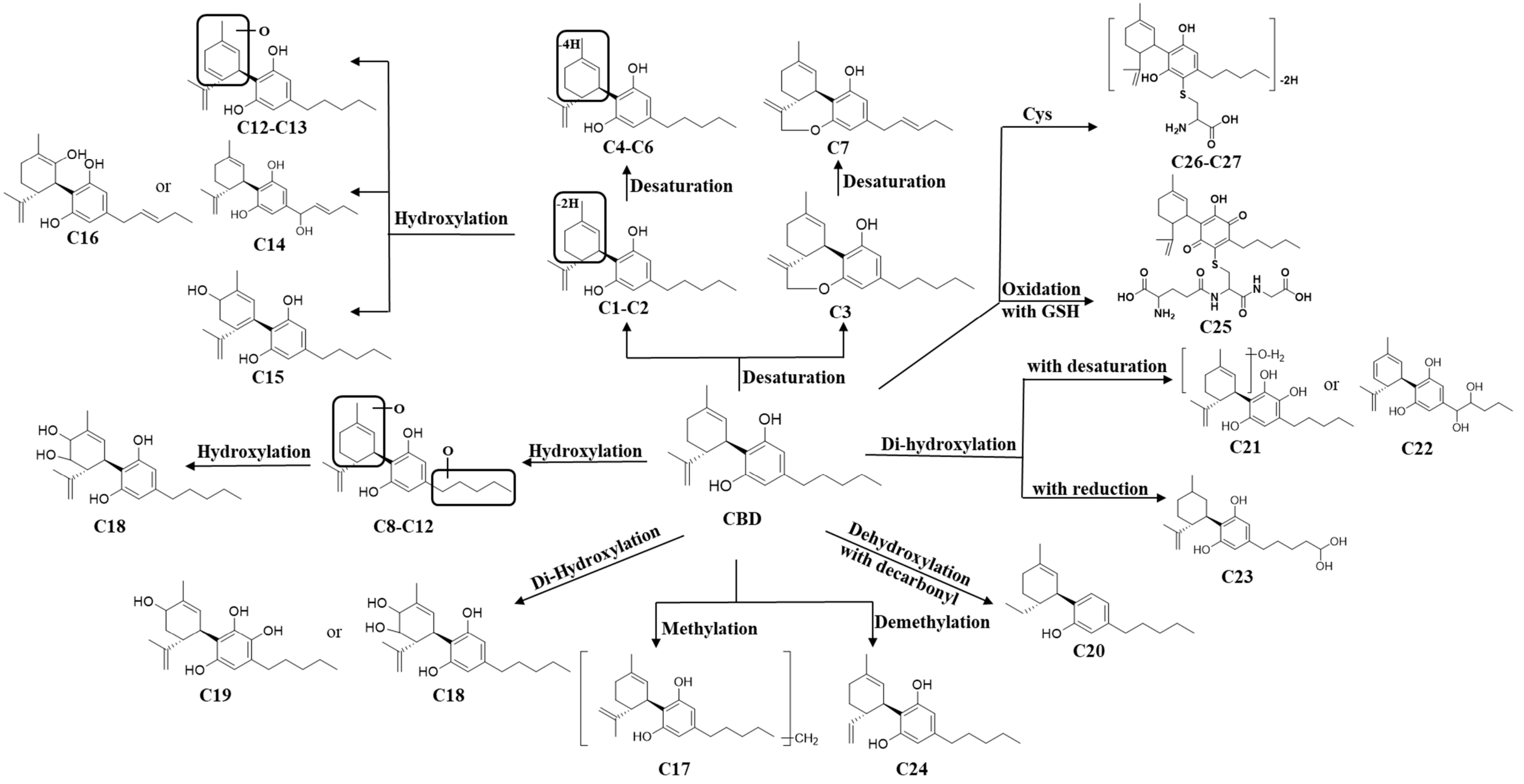

Table S1. Summary of CBD metabolites produced by the in vitro metabolism.

Number	RT (min)	Clog P	Observed *m/z* [M + H]	Delta ppm	Fragments	Predicted molecular formula	Identification	Source	NAPDH Independent
**C0**	8.19	6.64	315.2308	-5.04	93,135,193,259,273,315	$C_{21}H_{30}O_2$	CBD	HLM, MLM	-
***C1**	5.42	6.22	313.2154	-4.21	95,133,193,201,271,297,313	$C_{21}H_{28}O_2$	CBD-2H	HLM, MLM	NAPDH
***C2**	5.88	6.79	313.2174	2.11	95,133,201,271,313	$C_{21}H_{28}O_2$	CBD-2H	HLM, MLM	NAPDH
***C3**	8.45	6.99	313.2129	-2.68	93,135,191,219,257,301,313	$C_{21}H_{28}O_2$	CBD-2H	HLM	-
**C4**	4.57	6.33	311.1999	-3.98	133,213,255,269,311	$C_{21}H_{26}O_2$	CBD-4H	MLM	NAPDH
***C5**	4.85	-	311.2009	-0.55	133,255,213,255,311	$C_{21}H_{26}O_2$	CBD-4H	MLM	NAPDH
***C6**	6.47	6.49	311.1996	-4.82	91,181,198,216,269,311	$C_{21}H_{26}O_2$	CBD-4H	MLM	-
***C7**	7.85	6.51	311.1998	-4.18	109,195,231,269,283,311	$C_{21}H_{26}O_2$	CBD-4H	HLM	NADPH
**C8**	5.46	4.58	331.2261	-3.74	93,133,193,271,313,331	$C_{21}H_{30}O_3$	CBD+O	HLM, MLM,	NAPDH
**C9**	5.75	4.75	331.2263	-3.05	93,135,193,257,313,331	$C_{21}H_{30}O_3$	CBD+O	HLM, MLM	NAPDH
**C10**	7.04	4.85	331.2264	-2.75	93,135,197,313,331	$C_{21}H_{30}O_3$	CBD+O	HLM, MLM,	NAPDH
**C11**	7.45	4.88	331.2258	-4.56	93,109,135,193,205,271,313,331	$C_{21}H_{30}O_3$	CBD+O	MLM	-
**C12**	4.57	4.43	329.2114	-0.91	95,133,191,213,255,269,313	$C_{21}H_{28}O_3$	CBD+O-2H	HLM, MLM	NAPDH
**C13**	4.75	4.53	329.2101	-4.86	95,191,213,255,311,329	$C_{21}H_{28}O_3$	CBD+O-2H	HLM, MLM	NAPDH
**C14**	5.11	4.6	329.2114	-0.79	133,209,241,265,283,311,329	$C_{21}H_{28}O_3$	CBD+O-2H	HLM, MLM	NAPDH
**C15**	5.91	4.91	329.2101	-4.89	81,135,206,273,311,329	$C_{21}H_{28}O_3$	CBD+O-2H	HLM, MLM,	NAPDH
**C16**	7.85	5.46	329.2102	-4.43	109,131,193,243,271,301,329	$C_{21}H_{28}O_3$	CBD+O-2H	MLM	NAPDH
***C17**	8.54	-	329.2106	-3.22	107,133,209,255,271,287,329	$C_{21}H_{28}O_3$	CBD+CH_2	MLM	-
**C18**	4.57	3.51	347.2216	-1.81	95,133,191,255,311,329,347	$C_{21}H_{30}O_4$	CBD+2O	HLM, MLM	NAPDH
**C19**	6.64	3.95	347.2209	-3.80	107,193,193,205,271,311,347	$C_{21}H_{30}O_4$	CBD+2O	MLM	
***C20**	10.66	7.59	287.2367	-2.72	105,203,243,269,287	$C_{20}H_{30}O$	CBD-C-O	HLM, MLM	-
**C21**	4.68	-	345.2053	-3.71	191,226,271,327,345	$C_{21}H_{28}O_4$	CBD+2O-2H	HLM, MLM	NAPDH
**C22**	4.31	3.02	345.2077	3.19	95,133,213,255,267,309,327,345	$C_{21}H_{28}O_4$	CBD+2O-2H	MLM	NAPDH
**C23**	6.18	4.30	349.2372	-2.00	133,175,191,275,303,331,349	$C_{21}H_{30}O_4$	CBD+2O+2H	HLM	NAPDH
**C24**	4.72	6.24	301.2179	3.98	95,133,171,197,209,265,283,301	$C_{20}H_{28}O_2$	CBD-CH_2	MLM	NAPDH
**C25**	4.73	2.70	634.2788	-1.58	359,402,505,559,634	$C_{31}H_{43}N_3O_9S$	CBD+O-2H+GSH	HLM/MLM+GSH	-
***C26**	5.20	-	432.2202	-1.39	193,311,327,432	$C_{24}H_{33}NO_4S$	CBD-2H+Cys	HLM/MLM+Cys	NAPDH
***C27**	5.64	-	432.2204	-0.93	193,311,327,432	$C_{24}H_{33}NO_4S$	CBD-2H+Cys	HLM/MLM+Cys	NAPDH

* Indicates novel metabolites found in this study.

C0, Cannabidiol; +O, hydroxylation; -H_2, desaturation; +O-2H, hydroxylation with desaturation; -CH_2, demethylation; -C-O, devinylation with dihydroxylation; GSH, glutathione; Cys, Cysteine.

Table S2. Roles of CYPs in the formation of CBD metabolites.

	CYP1A1	CYP1A2	CYP1B1	CYP2A6	CYP2B6	CYP2C19	CYP2C8	CYP2C9	CYP2D6	CYP2E1	CYP3A4	CYP3A5	CYP4A11
**C1**			0.35	0.20	0.08	85.02	2.58	6.56	2.76	0.03	0.74	1.63	0.05
**C2**			0.11	0.77	0.54		3.05		80.66	0.67	3.01	6.70	4.49
**C3**			16.78	16.34	16.46		10.08			15.52	11.70		13.13
**C4**											44.62		55.38
**C5**									17.68		15.14	48.28	18.90
**C6**									10.44		7.63	75.22	6.71
**C7**									6.17		13.04	76.96	3.83
**C8**			1.30			4.86	15.71		71.67		0.07	6.31	0.08
**C9**	16.23	5.59	5.05		0.04	8.77	5.26	26.43	8.97	0.07	8.00	14.67	0.90
**C10**			5.82		1.42		5.12			2.74	26.39	50.35	8.17
**C11**	8.44	4.09	8.83	8.98	18.53		8.44	7.19		8.36	8.92	10.32	7.90
**C12**						23.11	1.74	0.87	74.27				
**C13**						19.92	7.23		72.84				
**C14**						84.67	15.33						
**C15**						62.48	3.99		33.53				
**C16**							100.00						
**C17**							100.00						
**C18**						25.23	2.10		72.67				
**C19**				11.13		37.64	11.15	12.20			13.49	14.38	
**C20**				73.80							26.20		
**C21**		0.70				51.39			45.77		0.89	1.26	
**C22**		6.34			5.68	48.75			27.83				11.40
**C23**			8.05	15.41	17.22	11.95					9.07	23.73	14.57
**C24**											35.34	37.90	26.76

c-DNA-expressed CYPs (Control, CYP1A1, CYP1A2, CYP2B6, CYP2C19, CYP2C8, CYP2C9, CYP2D6, CYP2E1, CYP3A4, CYP3A5, CYP4A11) were used to detect the roles of in individual CYPs in CBD metabolism. All samples were analyzed using UHPLC Q-Exactive MS. All data are expressed as a mean (n = 3). The numbers in the table are percentages, and they represent the percentage of the total of each metabolite formed across all cytochrome P450 enzymes that were tested.

Table S3. Molecular docking results (kcal/mol) estimated for the THC metabolites.

Name	-CDOCKER energy	-CDOCKER INTERACTION energy	Binding energy
		**THC metabolites**	
**T0**	8.27	47.84	-64.74
**T1**	-5.85	46.87	-60.78
**T2**	6.30	50.47	-32.71
**T3**	-15.77	44.56	-58.33
**T4**	-12.71	45.64	-65.17
**T5**	-6.86	47.33	-32.04
**T7**	34.45	49.35	-77.25
**T8**	10.41	48.86	-54.38
**T9**	8.67	48.59	-86.15
**T11**	5.62	53.26	-62.68
**T12**	17.18	48.08	-48.56
**T13**	9.08	48.39	-72.80
**T14**	14.07	48.21	-47.25
**T15**	11.32	49.46	-56.30
**T16**	6.18	51.16	-62.15
**T17**	10.61	50.43	-85.34
**T19**	14.86	54.83	-42.73
**T20**	31.47	51.43	-63.62
**T21**	11.20	53.40	-42.75
**T22**	3.06	45.34	-58.68
**T23**	16.00	49.99	-68.53
**T24**	8.59	56.09	-62.62
**T25**	13.16	52.13	-53.10
**T26**	20.31	54.34	-62.54
**T27**	13.73	52.52	-72.63
**T29**	30.50	81.27	-130.96
**T30**	8.93	69.35	-17.62
**T31**	6.94	55.09	-47.54
**T32**	7.87	57.20	-74.59

Table S4. Molecular docking results (kcal/mol) estimated for the CBD metabolites.

Name	-CDOCKER energy	-CDOCKER INTERACTION energy	Binding energy
		**CBD metabolites**	
**C0**	-1.98	45.16	-45.72
**C1**	-3.83	43.51	-47.45
**C2**	-6.22	48.04	-71.25
**C3**	-7.32	44.21	-42.78
**C4**	18.91	44.66	-61.01
**C6**	-11.98	45.83	-14.69
**C7**	-26.74	45.73	-44.86
**C8**	0.54	48.33	-24.69
**C9**	1.88	47.53	-24.67
**C10**	-2.93	47.14	-56.75
**C11**	-6.72	41.79	-33.38
**C12**	-6.82	46.32	-46.95
**C13**	-7.52	46.31	-70.70
**C14**	-20.18	41.45	-50.91
**C15**	0.89	49.13	-30.12
**C16**	-20.76	42.64	-38.33
**C18**	-9.30	45.10	-52.87
**C19**	0.65	49.22	-71.85
**C20**	16.12	46.39	-60.16
**C22**	-6.58	44.16	-57.33
**C23**	17.18	52.03	-94.77
**C24**	-26.74	45.73	-44.86
**C25**	-21.86	67.76	-11.70
